# Letter by the ISCB President

**DOI:** 10.1093/bioadv/vbab002

**Published:** 2021-05-12

**Authors:** Christine Orengo

**Affiliations:** President of the International Society for ComputationalBiology

This year is the 21st anniversary of the founding of the International Society for Computational Biology (ISCB) and the launch of *Bioinformatics Advances* is an exciting opportunity to mark this occasion.

ISCB has partnered with publishers and affiliated with journals in the past and is continuing some of these partnerships. We feel that now the time has come to start our first society-co-owned journal.

Since the foundation of ISCB, the field has grown far beyond our expectations with many more biologists becoming involved in computational work either directly or through increasingly interdisciplinary collaborations. Furthermore, the success and acceleration of experimental technologies including sequencing, imaging, proteomics, single-cell analysis and Cryo-Electron microscopy, amongst others, has driven our computational methods forward tremendously. Today the impact of ‘big data’ in biology is explicitly recognized and prioritized by funding agencies and even reported in national newspapers. Our computational methods are being enhanced by the increased scale and quality of these data and, in turn, the advances in our methods enable us to mine these data much more effectively. The success of DeepMind’s recent Artificial Intelligence foray into protein structure prediction was reported widely in newspapers across the globe. Other advanced machine learning strategies are also making significant impacts in many diverse areas of biology. Our tools, resources and analysis strategies are likely to bring many pioneering insights into biological systems in the coming years and decades.

Since our computational community is very broad with research covering methods advancement and data analyses yielding novel biological insights, the aims of this new journal are to capture this breadth and balance contributions from across the spectrum, making the developments accessible to an even wider audience of biologists. In addition, the pace at which new technologies are emerging has increased—both regarding experimental and computational technologies—and this new journal will aim to report these exciting new technologies and capture the perspectives they bring to our field. Finally, the Society is keen to increase initiatives that enhance equality and representation across our diverse populations. The Society is very pleased to see that the journal aims to be a forum that captures the diversity of our scientists, whether it be with respect to scientific background, gender, seniority, ethnicity or geographical region.

We believe that *Bioinformatics Advances* will rapidly become another leading journal in the area of computational biology and, as an official journal of ISCB, will be an important venue in which ISCB members will publish their findings and learn of the work of others, both ISCB members and nonmembers.

The ISCB believes that open access to scientific research is an important means of more rapidly disseminating scientific advances, and of communicating the findings to scientists around the world. Open access provides distinct advantages to scientific authors, including greater freedom to reuse and republish text, and greater availability of text for electronic data mining. In combination with rapid publication and high editorial standards, these provide compelling reasons for computational biologists to read and publish in *Bioinformatics Advances*.

In choosing Oxford University Press (OUP) as the publisher of the official journal of the society, ISCB was also guided by the quality of OUP, its board of directors and senior editorial staff. It has been, and will continue to be, a pleasure collaborating with such an experienced and highly skilled group. OUP brings great expertise in an experienced editorial staff and in developing the open-access publishing model. The experience and expertise of OUP ensure that *Bioinformatics Advances* meets the highest production standards. Scientific leadership from ISCB, including the appointment of Thomas Lengauer, past president of ISCB and Alex Bateman, a Vice President of ISCB as editors-in-chief, will ensure the highest scientific standards. The Society and the journal thus work together to strengthen our community. The participation of ISCB members will be critical to the success of *Bioinformatics Advances*. As authors, contributors, reviewers and editorial board members, the members of ISCB will work with OUP in building a successful journal. Together, ISCB and OUP make ideal partners, and I am sure that you will join me in eagerly awaiting the first issues of *Bioinformatics Advances*.

